# Tissue Factor in Dermatitis Herpetiformis and Bullous Pemphigoid: Link between Immune and Coagulation System in Subepidermal Autoimmune Bullous Diseases

**DOI:** 10.1155/2015/870428

**Published:** 2015-12-29

**Authors:** Agnieszka Zebrowska, Malgorzata Wagrowska-Danilewicz, Marian Danilewicz, Joanna Wieczfinska, Ewa Pniewska, Michal Zebrowski, Elzbieta Waszczykowska, Anna Wozniacka, Makandjou-Ola Eusebio, Miroslawa Pietruczuk, Rafal Pawliczak

**Affiliations:** ^1^Department of Dermatology and Venereology, Medical University of Lodz, Hallera Square 1, 90-497 Lodz, Poland; ^2^Laboratory of Nephropathology, Medical University of Lodz, Pomorska 251 Street, 92-213 Lodz, Poland; ^3^Department of Immunopathology, Faculty of Biomedical Sciences and Postgraduate Training, Medical University of Lodz, Zeligowskiego 7/9, 90-752 Lodz, Poland; ^4^Department of Social Medicine, Medical University of Lodz, Zeligowskiego 7/9, 90-752 Lodz, Poland; ^5^Department of Laboratory Diagnostics, Medical University of Lodz, Kopcinskiego 22, 90-153 Lodz, Poland

## Abstract

Dermatitis herpetiformis (DH) and bullous pemphigoid (BP) are skin diseases associated with eosinophilic and neutrophilic infiltrations. Although chemokines are critical for the selective accumulation and activation of various leukocyte subsets in the inflammatory process, there are few findings concerning inflammatory cells and production of coagulation factors in blistering diseases. Skin biopsies were taken from 14 patients with DH, 27 with BP, and 20 control subjects. The localization and expression of tissue factor (TF) in skin lesions and perilesional skin were studied by immunohistochemistry and confirmed by Western Blot. Moreover the plasma concentrations of TF were measured by immunoassays. D dimers, fibrinogen, and selected coagulation parameters were measured by routine methods. Expression of TF in the epidermis and in inflammatory influxed cells in dermis was detected in skin biopsies from BP patients. Examined TF expression was detected in perilesional skin of all BP patients too. The expression of TF was not observed in biopsies from healthy people and DH patients. The findings of the study show an increased expression of tissue factor in the lesional and perilesional skin of patients with bullous pemphigoid. The difference in chemokine pattern expression and variations in the cellular infiltration in BP and DH cause variable expression of TF.

## 1. Introduction

Dermatitis herpetiformis (DH) is one of the subepidermal autoimmune bullous diseases (ABD) characterized by skin and intestinal lesions. Skin lesions include polymorphic eruption accompanied by severe pruritus [1]. Diagnosis of DH is established based on the results of direct immunofluorescence test (DIF) and the presence of circulating IgA antibodies directed against endomysium and/or tissue and epidermal transglutaminase (tTG, eTG) [2, 3]. Skin lesions in DH are histologically characterized by neutrophilic infiltrate leading to destruction of basement membrane zone (BMZ) proteins, anchoring fibers, and blister formation [4–6].

Bullous pemphigoid (BP) is a blistering disease, characterized by inflammatory infiltrate in the dermis, presence of IgG and C3 deposits along the basement membrane zone, and circulating IgG autoantibodies. Autoantibodies binding to autoantigens (BPAG1 and BPAG2) localized in the basement membrane of the epidermis activate a series of immunological and enzymatic phenomena leading to destruction of BMZ components and anchoring fibers and blister formation as in DH [7, 8].

Ultrastructural studies confirmed also the presence of intensive inflammatory infiltrate at dermoepidermal junction, as well as destruction of components of extracellular matrix in BP and DH [9–11].

In the available literature, there are few reports on the expression of procoagulant factors, abnormal activation of coagulation process, and their role in the creation of blisters. Recently, the dominant role of eosinophils in the inflammatory infiltrate in bullous diseases is postulated. Therefore, the fact that they might be an important source of procoagulant tissue factor (TF) gives the scientific basis to consider its contribution of this process in the pathogenesis of lesions in the subepidermal bullous diseases.

Recent studies have shown an increased risk of fatal thrombotic events in patients with BP treated with glucocorticoids [12]. Confirmation of these complications is the increased concentration of D dimers and prothrombin in the serum of patients in the active stage of the disease [13]. Therefore, it is important to define the role of procoagulant factors in the destruction of the basement membrane in pemphigoid and activity of eosinophils and mediators of these cells in skin lesions and normal looking skin as well as in serum in the active phase of the disease.

Under physiological conditions, there is a balance between the coagulation and fibrinolysis, but in pathological states this balance might be deteriorated. In bullous diseases inflammation disrupts homeostatic system and moves it towards the prothrombotic status. Rico et al. [14] describe the dominance of the Th2 cytokine profile in blistering diseases. Interleukin-5, produced mainly by Th2 lymphocytes, is a key factor in the differentiation and activation of eosinophils [14–16].

Inflammatory cells are important factors in the coagulation system. Eosinophils, the dominant inflammatory cells in BP, seem to be a major source of intravascular tissue factor [17]. TF is the primary activator of extrinsic coagulation pathway [17, 18].

Coagulation pathway might cause the activation of adhesion molecules expression and subsequent release inflammatory mediators and proteolytic enzymes [10, 19]. Recent experimental studies have also shown that these processes may also activate procoagulant factors that cause the development of blood clots, which are the most common cause of complications in patients with BP [20].

TF, in addition to well-documented prothrombotic properties, plays an important role in the inflammatory process. Constitutive expression of TF is present on fibroblasts and epithelial cells, while on monocytes and macrophages it is induced by antigen-antibody complexes and complement activation products and proteins as well as by inflammatory cytokines [21, 22].

The goal of this study was to assess the activity of procoagulant factors and their impact on the inflammatory infiltrate in BP and DH before treatment. The data might be an important therapeutic indication allowing for alleviating the high mortality rate among patients with pemphigoid treated with corticosteroids and confirming a new trend in the treatment of BP.

## 2. Materials and Methods

### 2.1. Patients

The study group involved 41 patients, including 27 patients with BP (15 women and 12 men, age range: 58 to 84 years, mean age: 68.5 years) and 14 patients with DH (6 women and 8 men, age range: 18 to 70 years, mean age: 49.8 years) in the active phase of the disease. The control group consisted of 20 healthy subjects (5 women and 5 men, age range: 50–80 years, mean age: 71.6 for BP patients and 6 women and 4 men, age range: 19–49 years, mean age: 42.0 years for DH patients). In DH patients direct immunofluorescence tests revealed the presence of granular deposits of IgA in skin papillae and indirect immunofluorescence tests were positive for IgAEmA in all the patients (titer 1 : 40–1 : 640, median 1 : 80). Anti-tissue transglutaminase antibodies measured using an immunoassay were present in 7/10 cases (median: 5.1 IU/mL (range: 0.0–186.3; IU/mL)).

The BP patients were at an active stage of the disease and the average score on BPDAI (Bullous Pemphigoid Disease Activity Index) was 38 ± 11. The histopathologic findings according to Ackerman in all cases were fully developed. In all the patients direct immunofluorescence test revealed IgG/C3 linear deposits along BMZ. In salt split test deposits were observed in epidermal part of the blister. Using indirect immunofluorescence test circulating IgG antibodies were found in 9/14 patients. ELISA test showed the anti-NC16 autoantibodies present in serum of 11 out of 14 patients.

All the patients signed informed consent before entering the study and the study protocol (#RNN/93/03/KE) was approved by The Local Ethical Committee of Medical University of Lodz. From all patients in the study group and the healthy control group the blood was collected to several samples. In all patients the blood was collected during active stage of disease without any administered treatment. Skin biopsies from skin lesions were taken for histopathological examination in the study groups and from the healthy skin in the neck area in control group.

### 2.2. Immunohistochemistry

Paraffin-embedded sections were used for routine H+E staining and for immunohistochemistry with DAKO EnVision detection system using immunoperoxidase method. The following primary monoclonal antibodies were used: anti-tissue factor obtained from American Diagnostica Inc., Greenwich, CT, USA, according to manufacturers' instruction.

### 2.3. Morphometry

Histological morphometry was performed by means of image analysis system consisting of a PC computer equipped with a Pentagram graphical tablet, Indeo Fast card, produced by Indeo (Taiwan), and color TV camera Panasonic (Japan) coupled to a Carl Zeiss microscope (Germany). The TF positive cells were counted in a sequence of 7–10 consecutive computer images of 400x high power fields, 0.0047 mm^2^ each. The results were expressed as percentages of TF positive cells of all lymphocytes determined by their morphology.

### 2.4. Immunoassay

TF levels were measured in plasma in all patients and healthy controls undergoing skin biopsy. Five mL of venous blood was drawn from the ulnary vein and after centrifugation serum was stored at −20°C for an immunoassay. The enzyme-linked immunoassays were used to measure TF-IMUBIND Tissue Factor ELISA Kit (American Diagnostica Inc.). D dimers levels were performed, using the optical test STA Compact (Roche Diagnostics); fibrinogen levels were measured using chronometric method of Clauss and turbidity method; coagulogram was done according to the standard procedure; DD (D dimers), F2 (factor II), F5 (factor V), F7 (factor VII), and venom (snake venom factor V activator) were assessed by Destiny Plus in BP patients (Horiba ABX).

### 2.5. Immunoblotting

Total protein from frozen skin samples was extracted in RIPA protein extraction buffer, supplemented with protease inhibitor cocktail (Sigma-Aldrich, St. Louis, MO, USA). The lysate was centrifuged at 14,000 ×g for 20 min and the pellet was discarded. Protein concentrations were determined by the BCA Protein Assay Kit (Pierce Thermo Scientific, USA). Electrophoresis was performed in SDS-NuPAGE Gels (Life Technologies, California), subsequently transferred to a nitrocellulose. The membrane was blocked and incubated with the mouse monoclonal anti-TF (Merck Millipore, Billerica, USA). Afterwards the membrane was washed with TBST and incubated in PBST containing the goat anti-mouse IgG secondary antibodies conjugated with alkaline phosphatase (Merck Millipore, USA). The bands were developed using BCIP/NBT Alkaline Phosphatase Substrate (Merck Millipore, Billerica, USA), analyzed with ImageJ 1.34s software (Wayne Rasband, National Institutes of Health, Bethesda), and expressed as optical density (OD).

### 2.6. Statistical Methods

All values were expressed as mean ± SEM. Differences between groups were tested using unpaired Student's *t*-test proceeded by the evaluation of normality. The Mann-Whitney *U* test was used where appropriate. Results were considered statistically significant if *p* < 0.05. The data of immunoblotting were analyzed with Student's *t*-test and* Mann-Whitney U* test.* Levene's test* was used to assess homogeneity of variances. The level of significance was defined as *p* < 0.05.

## 3. Results

### 3.1. Immunohistochemistry: Tissue Factor

**Figure 1:**
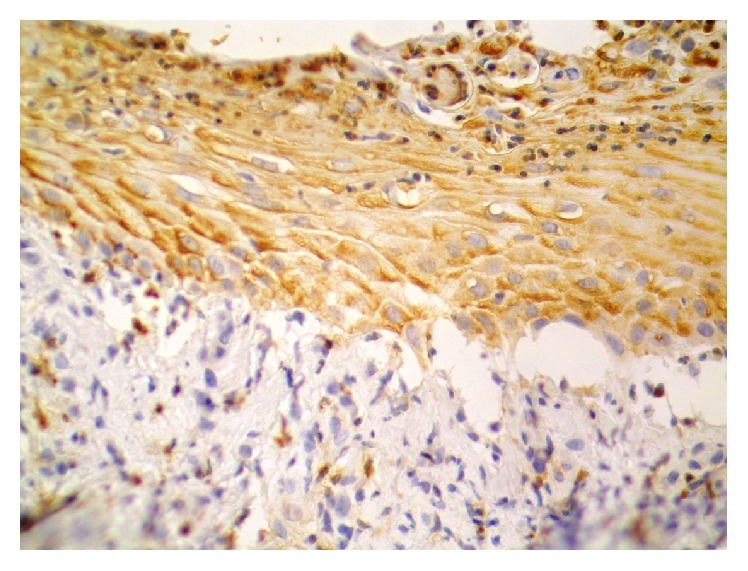
Expression of TF in epidermis and cells of influx. Skin lesions. Bullous pemphigoid. 400x Immunohistochemistry.

**Figure 2:**
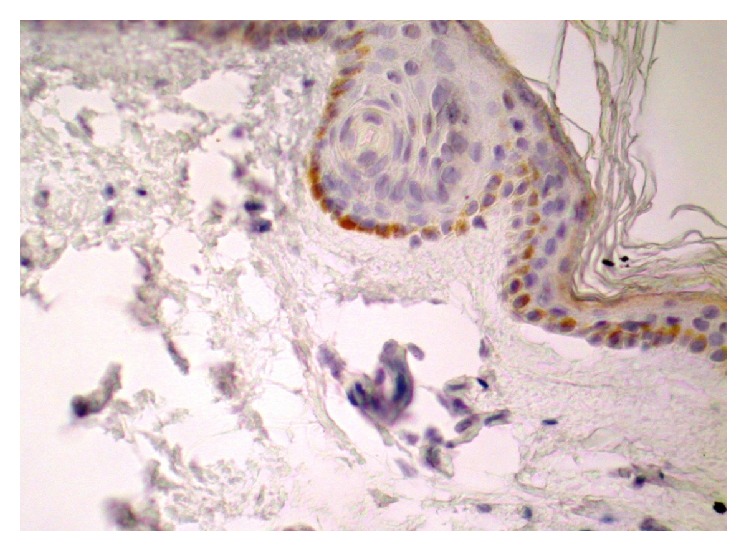
Expression of TF in epidermis. Perilesional skin. Bullous pemphigoid. 400x. Immunohistochemistry.

TF expression was demonstrated in cells in the inflammatory infiltrates (eosinophils) and keratinocytes and in the blister fluid (Figure 1). In patients with BP, the expression of TF was also shown in the perilesional skin (in 17 out of 27 individuals) (Figure 2). In patients with BP, in skin lesions, a high expression of TF has been demonstrated mainly in keratinocytes of the basal layer (in 20 out of 27 biopsies) and also in other layers of the epidermis (in 19 out of 27 individuals), but expression insensitivity was lower.

**Figure 3:**
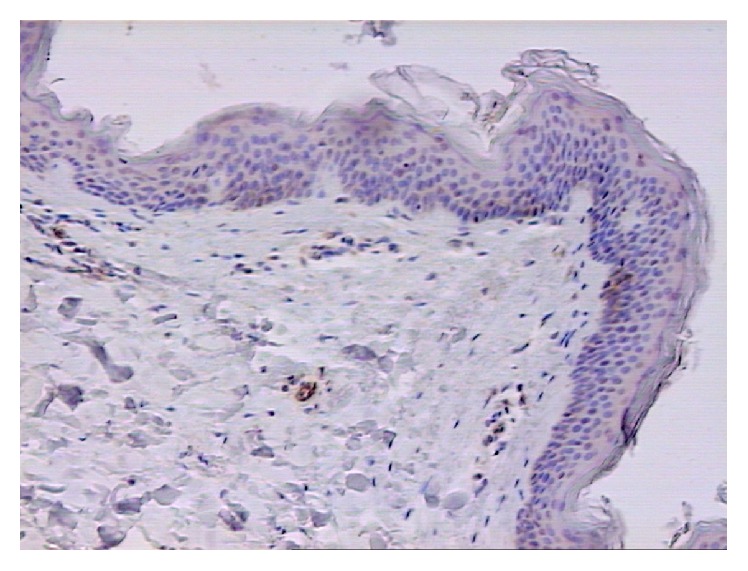
Lack of expression of TF. Healthy skin. 100x. Immunohistochemistry.

Morphometric analysis showed a higher expression of TF in skin lesions than in perilesional skin in patients with BP (*p* < 0.02) (Table 1). Studies of patients with DH did not show a significant expression of TF either in pathologically changed tissues (TF was present in 3 out of 14 samples) or in perilesional skin (Figure 3). TF protein expression was also found in biopsy specimens from healthy individuals (in 2 out of 14). Therefore, the TF expression, in healthy group, was significantly lower than in the lesions in BP and comparable to expression in DH lesions.

### 3.2. Immunoblotting

**Figure 4:**
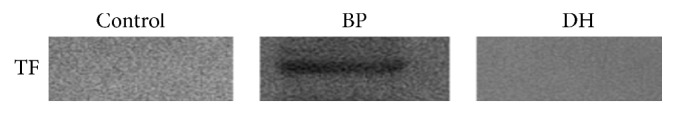
Tissue factor protein immunoblotting.

**Figure 5:**
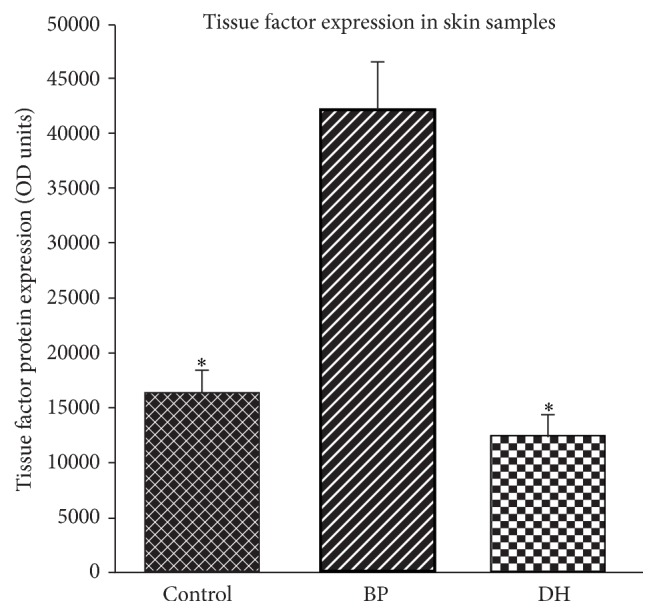
Mean value of optical densities (OD) of control and DH background and BP protein. Asterisks indicate a significance difference (^*∗*^ *p* < 0.05) (mean ± SEM), TF: tissue factor; DH: Duhring disease; BP: bullous pemphigoid.

Utilizing an immunoblotting technique and specific antibodies against TF we found significantly higher expression of TF in BP patients as compared to DH patients (*p* < 0.05) and as compared to healthy controls (*p* < 0.05) as shown in Figures 4 and 5.

### 3.3. Immunoassay

#### 3.3.1. Tissue Factor

The mean TF plasma level in BP patients was 847.2 ± 402.7 pg/mL as compared to patients with DH (285.7 ± 187.7 pg/mL) and in the control groups, 252.6 ± 169.3 pg/mL for BP and 221.9 ± 153.3 pg/mL for DH. These results indicate a statistically significant higher TF plasma concentration in patients with BP as compared to DH patients' and the control subjects (*p* < 0.001).

#### 3.3.2. D Dimers

Prior to treatment, the D dimers levels in patients with BP were within the upper limits of the reference values (in 20 out of 27 patients). The mean D dimers level was 0.49 ± 0.4 mg/mL (whereas the reference value range was 0.33–2.9 mg/mL). In 7 patients these levels were elevated and were between 1.2 and 2.9 mg/mL. These values were higher as compared to DH patients and the control group.

The mean D dimers values in DH patients were 0.31 ± 0.19 mg/mL and were within the reference value. In the control group of patients, used as a reference group for BP patients, D dimers levels were within normal limits (the mean for control group was 0.29 ± 0.3 mg/mL). In the control group of patients used as a reference group for patients with DH, D dimers levels were within normal limits; the mean was 0.34 ± 0.6 mg/mL. Therefore, there is a statistically significant difference in D dimers levels between the DH and BP patients (*p* < 0.05). There is also a statistically significant difference in D dimers levels between BP patients and the control group (*p* < 0.05).

### 3.4. Coagulogram

In BP patients coagulation parameters were as follows: prothrombin time mean of 13.1 ± 0.2 sec (reference value: 12–18 sec), mean prothrombin index of 85.9%  ± 0.8% (reference range: 70–100%), mean INR of 1.04 ± 0.4 (reference range 0.9–1.30), and mean APTT of 31 s ± 1.0 sec (reference range: 26–36 sec.). In patients with DH coagulation parameters were as follows: mean prothrombin time of 15.3 ± 0.5 sec, mean prothrombin index of 78.9%  ± 0.6%, mean INR values of 1.28 ± 0.3, mean APTT of 29 sec ± 1.0 sec (reference range: 26–36 sec), and average values of 29 s ± 1.0 sec.

In the control group used for comparison with BP patients, coagulation parameters were as follows: mean prothrombin time of 14.2 ± 0.2 sec, mean prothrombin index of 88.7%  ± 0.4, mean INR of 1.17 ± 0.5, and mean APTT of 30 sec ± 1.0 sec. In the control group used for comparison with DH patients, coagulation parameters were as follows: mean prothrombin time of 14.9 ± 0.5 sec, mean prothrombin index of 98.7%  ± 0.2, mean INR of 1.21 ± 0.4, and mean APTT of 28 sec. ± 1.0 sec.

There is no difference in coagulation parameters between BP and DH groups and examined groups and healthy individuals. The platelets levels in all groups were within the reference range (BP patients: 284.0 ± 96.9 × 10^9^/L; DH patients: 187.0 ± 58.4 × 10^9^/L; and control group: 254.0 ± 79 × 10^9^/L).

#### 3.4.1. Fibrinogen Levels

In BP patients, mean fibrinogen level was 4.50 ± 0.18 g/L as compared to 2.60 ± 0.3 g/L in the control group. DH patients mean fibrinogen concentration was 2.80 ± 0.14 g/L as compared to 2.45 ± 0.26 g/L in the control group. There is a statistically significant difference (*p* < 0.05) between the group of patients with DH and BP, as well as between BP patients and the control group (*p* < 0.05).

#### 3.4.2. DD, F2, F5, F7, and Venom Measurements by Destiny Plus System in BP Patients

**Figure 6:**
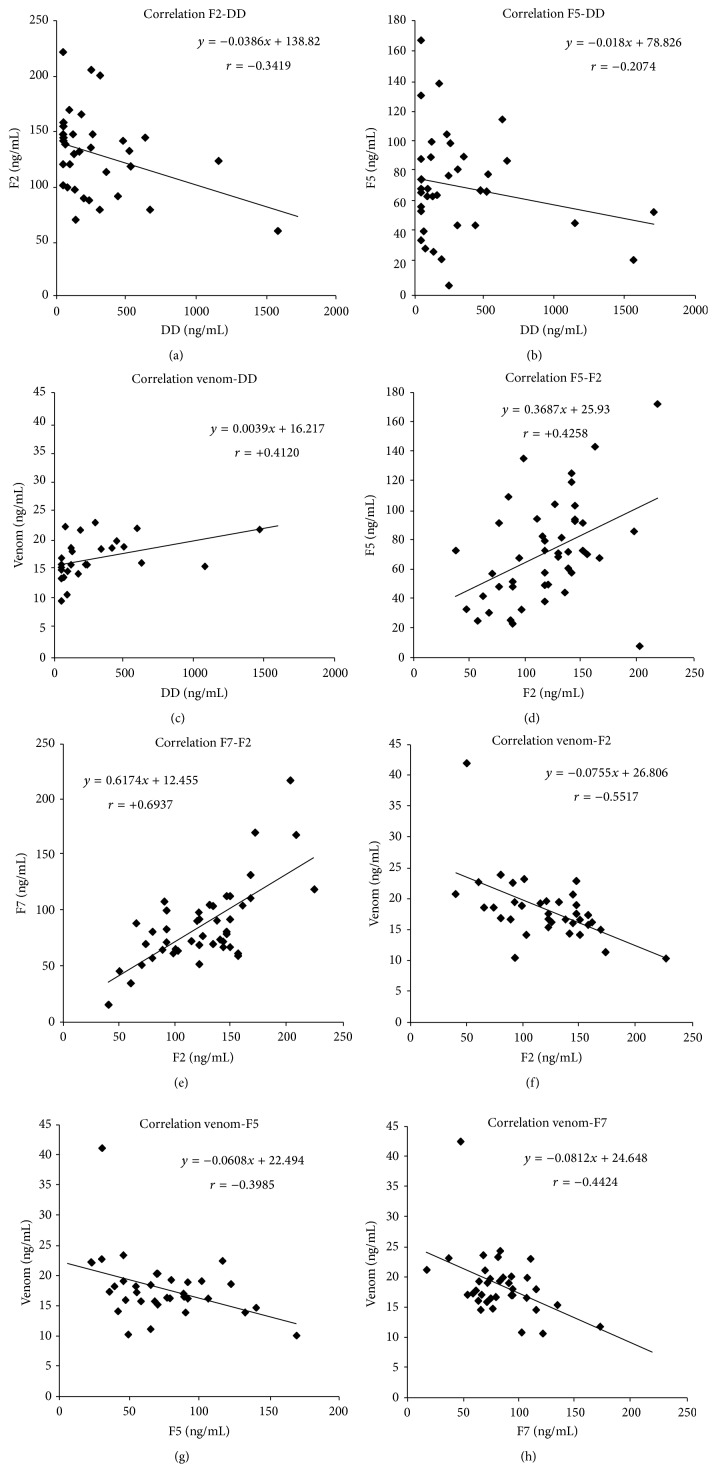
F2, F5, F7, D dimers, and venom: correlations in BP patients.

The D dimers levels were significantly higher in the BP patient group as compared to controls (312 ng/mL versus 96 ng/mL) (*p* < 0.01). All blood coagulation factors like F2, F5, F7, and venom in BP and DH patients and control group were within the reference values. The following correlations were found in BP patients between the following: F2 and DD (*r* = −0.3419, *p* = 0.003); F5 and DD (*r* = −0.2074, *p* = 0.005); DD and venom (*r* = +0.4120, *p* = 0.0111); F5 and F2 (*r* = + 0.4258, *p* = 0.0031); F7 and F2 (*r* = +0.6937, *p* = 0.00001). Similarly, they were found between the following: venom and F2 (*r* = −0.5517, *p* = 0.0001); venom and F5 (*r* = −0.3985, *p* = 0.0054); venom and F7 (*r* = −0.4424, *p* = 0.0016) (Figure 6).

## 4. Discussion

Eosinophils in response to many stimuli secrete many proteins including eosinophil cationic protein (ECP). Elevated ECP prolongs the clotting time, due to its antagonism with heparin and streptokinase [23]. Another protein, playing an important role in coagulation process, is tissue factor (TF), also produced by eosinophils. Glucocorticoids do not stop the release of these proteins [24], but immunosuppressive agents such as cyclosporine inhibit the secretion of ECP from eosinophils.

Marzano et al. [13] in their study evaluated the TF expression in skin biopsies in BP patients and showed that immunohistochemistry revealed strong TF reactivity in BP skin, and colocalization studies confirmed eosinophils as a source of TF. Our results indicate a statistically significant difference in the value of TF between the group of patients with DH and BP and BP and the control group.

Elevated levels of the prothrombin fragment F1 + 2 and both plasma and blister fluid of BP patients in the active phase of the disease were reported [13]. During the remission, the concentration of coagulation activation markers was normal. The concentration of the prothrombin fragment F1 + 2 correlated with the concentration of immunoglobulins directed against the BP180 antigen [13].

TF is the main activator of extrinsic coagulation pathway, which in addition to well-documented prothrombotic properties plays an important role in the inflammatory process [17]. It is a recognized factor connecting the immune system with coagulation system. Expression of TF present in fibroblasts, epithelial and endothelial cells, and macrophages is induced by antigen-antibody complexes, complement activation products, proteins, and proinflammatory cytokines [22]. Our study showed low level TF expression in DH patients and healthy subjects, confirming its constitutive production.

Cugno et al. [22] emphasized the role of TNF and IL-6, which are increased in BP, in the induction of TF expression. The eosinophilic infiltration in BP skin might provoke an elevation of TF concentration in this disease. Morphometric analysis showed a higher expression of TF in skin lesion than in perilesional skin in patients with BP opposite to DH and control biopsies. In our study, we confirmed that kind of influx has important role in activation of TF in skin.

The inflammatory and neoplastic processes of coagulation and fibrinolysis system are activated in response to the local infiltration of inflammatory cells and increased vascular permeability [18, 22]. TF increases the influx of monocytes to the sites of inflammation [25, 26], and its induction in monocytes may depend on a direct interaction with T cells. Several studies have confirmed the effect of the inflammatory process in the excessive production of tissue factor [27–30].

Both in the case of pemphigoid and DH in our earlier studies, we showed in skin lesions increased expression of proinflammatory cytokines which may activate the production of TF. However, only in BP, expression was increased in both: skin lesions and blood. This is probably related to eosinophilic infiltration in BP and production by these cells procoagulant factors [17].

The product of activation of TF, thrombin, acts by increasing vascular permeability for inflammatory cells and causing the release of interleukin production and activation of adhesion molecules [22]. In our earlier studies, we confirmed the increased expression of adhesins and MMPs, which may be an effect of action of TF in BP [31].

In the aspect of described results, participation of activation markers of eosinophils, mast cells activation, and coagulation factors in the pathogenesis of some subepidermal blistering diseases appears to be particularly interesting [25, 26].

Abnormal expression of procoagulant factors in the tissue may be responsible for the inflammatory process and blister formation in BP. According to literature [32, 33], up to 25% of the cells in the DH influx are eosinophils. In addition, myeloperoxidase, ECP, and major basic protein secreted by eosinophils were found in sera of patients with DH, which may indicate the production of inflammatory mediators by eosinophils in DH [33]. The small TF expression in our study in DH patients may be related to the fact that the presence of a small population of eosinophils in the inflammatory infiltrates in these patients.

This study showed that the group of BP patients revealed the normal mean D dimer concentration, however the individual results were within the upper limit of the reference range, and some patients had elevated D dimer levels. However, in the evaluation by the Destiny Plus System, D dimer concentration was significantly higher in the BP patient group as compared to controls. It should be noted that these values were determined prior to treatment with corticosteroids, which exacerbate prothrombotic processes. The values of D dimer in patients with DH were normal and similar to the levels in healthy subjects.

The treatment of patients with BP, especially the elderly, with local steroids or antihistamines and immunomodulators has been postulated [34, 35]. Therefore, it seems important to indicate the need to change the current treatment of patients with BP with the standard treatment of corticosteroid into drugs that do not increase the risk of thrombotic complications. It is important in planning treatment preventing complications.

In the therapy of pemphigoid, we use many drugs acting through different mechanisms. Because of the key role of autoimmune phenomena, most commonly used drugs are glucocorticosteroids (GCS), a class of drugs having a particularly strong impact on the homeostatic system [36]. Effects of thrombogenicity related to GCS might be connected with their direct influence on the coagulation system and the metabolic disturbances linked to the nature of the disease [37]. Our study also showed elevated levels of fibrinogen in patients with BP compared to the patient with DH and control group. Some clinical studies have shown that fibrinogen level above 3.5 g/L is seen as the value that causes an increased risk of thrombosis and thus an increased risk of ischemic heart disease or a stroke [38, 39]. The main cause of death in patients with pemphigoid is the complications of steroid treatment, which is considered to be the most effective in this disease [40–42]. Rzany et al. [43] underline that embolic complications with severe bacterial infections are the most common side effects in the course of corticosteroid overall in the age group above 70 years. Other authors also confirm the good results of local therapy in the treatment of bullous pemphigoid and reducing the incidence of adverse events compared with patients treated with oral steroids [44].

So far, there are no data on the expression of procoagulant factors and abnormal activation of coagulation process in the formation of blisters. However, the dominant role of eosinophils in the inflammatory infiltration in BP was recently revealed and the fact that they secrete tissue factor gives the scientific basis to consider the contribution of this process in the pathogenesis of lesions in the subepidermal bullous diseases. Demonstration of elevated D dimer and prothrombin in patients in the active phase of the disease indicates the need to assess the suitability of patients for thromboembolic complications. An increased risk of death due to the stroke in patients with BP treated with glucocorticoids might confirm these findings. Despite these risks, the latest consensus on the treatment of pemphigus and of bullous pemphigoid still recommends the use of corticosteroids in the treatment of BP [45].

Our results may be useful in clinical practice and objective determination of the degree of disease activity as well as an important therapeutic indication which allows reducing the high mortality rate among patients with pemphigoid treated with corticosteroid and confirming a trend in the treatment of this disease with antihistamines and nonsteroidal immunomodulators.

## Figures and Tables

**Table 1:** Morphometric analysis of TF expression in skin lesions and in perilesional skin (in keratinocytes and infiltrates) in patients with BP. Immunohistochemistry.

	Mean ± SEM
BP skin lesions (*n* = 27)	1.34 ± 0.62
BP perilesional skin (*n* = 27)	0.92 ± 0.54
Control group (*n* = 10)	0.16 ± 0.28
DH skin lesions (*n* = 14)	0.18 ± 0.24
DH perilesional skin (*n* = 14)	0.15 ± 0.22

BP skin lesions versus BP perilesional skin	*p* < 0.02
BP skin lesions versus control group	*p* < 0.001
DH skin lesions versus perilesional skin DH	ND
BP skin lesions versus DH skin lesions	*p* < 0.05
BP perilesional skin versus DH perilesional skin	*p* < 0.05
